# Fine Tuning Non-invasive Respiratory Support to Prevent Lung Injury in the Extremely Premature Infant

**DOI:** 10.3389/fped.2019.00544

**Published:** 2020-01-10

**Authors:** Kirsten Glaser, Christian P. Speer, Clyde J. Wright

**Affiliations:** ^1^University Children's Hospital, University of Würzburg, Würzburg, Germany; ^2^Section of Neonatology, Department of Pediatrics, University of Colorado School of Medicine, Children's Hospital Colorado, Aurora, CO, United States

**Keywords:** preterm infant, respiratory distress syndrome (RDS), lung injury, bronchopulmonary dysplasia (BPD), non-invasive ventilation, non-invasive respiratory support, continuous positive airway pressure (CPAP), sustained lung inflation (SLI)

## Abstract

Within the last decades, therapeutic advances, such as antenatal corticosteroids, surfactant replacement, monitored administration of supplemental oxygen, and sophisticated ventilatory support have significantly improved the survival of extremely premature infants. In contrast, the incidence of some neonatal morbidities has not declined. Rates of bronchopulmonary dysplasia (BPD) remain high and have prompted neonatologists to seek effective strategies of non-invasive respiratory support in high risk infants in order to avoid harmful effects associated with invasive mechanical ventilation. There has been a stepwise replacement of invasive mechanical ventilation by early continuous positive airway pressure (CPAP) as the preferred strategy for initial stabilization and for early respiratory support of the premature infant and management of respiratory distress syndrome. However, the vast majority of high risk babies are mechanically ventilated at least once during their NICU stay. Adjunctive therapies aiming at the prevention of CPAP failure and the support of functional residual capacity have been introduced into clinical practice, including alternative techniques of administering surfactant as well as non-invasive ventilation approaches. In contrast, the strategy of applying sustained lung inflations in the delivery room has recently been abandoned due to evidence of higher rates of death within the first 48 h of life.

## Introduction

Bronchopulmonary dysplasia (BPD) is the most prevalent complication related to prematurity. It is associated with an increased risk of mortality, as well as multiple in-hospital and post-discharge morbidities (1, 2). Considerable advances in neonatal strategies and corresponding improvements in survival from respiratory distress syndrome (RDS) have altered the nature of BPD, but have not changed its incidence in extremely premature preterm infants (3). Apparently, practice change to prevent neonatal lung injury has not been effective or has not evolved quickly enough in this population of infants. The pathogenesis of BPD is multifactorial, and involvement of various underlying mechanisms affecting immature airway structures leads to inflammation, apoptosis and extensive extracellular matrix remodeling, culminating in pathologic alveolarization and angiogenesis. It is well-established that exposure to and duration of invasive mechanical ventilation (IMV) and the resulting volutrauma, barotrauma, atelectrauma, rheotrauma, and biotrauma are major contributing factors (4–6). This causal relationship was first noted in the 1970's (7, 8). Unfortunately, episodes of IMV often cannot be avoided for the highest risk babies, and exposure to IMV may even remain the rule rather than the exception in some countries. In the United States based NICHD Neonatal Research Network, in 2012, 82% of all infants who were born between 22 and 28 weeks' gestation and survived more than 12 h were mechanically ventilated during their NICU stay (3). While refinements in ventilators and modes of ventilation have been introduced to minimize lung injury, no data exist that definitely prove any one mode of ventilation or any one ventilator beneficial (9–11).

## CPAP and the Physiologic Basis for Supporting Functional Residual Capacity as a Means to Prevent Mechanical Ventilation

Successful transition to postnatal life requires the opening and aeration of the lung. This process is impaired in many extremely preterm infants (12). Multiple unique physiologic and anatomic features put the tiny baby at risk of having a low functional residual capacity (FRC). Any degree of surfactant deficiency will bias the lung toward atelectasis. The structural immaturity and increased compliance of the chest wall dictate impaired stability of those structures needed for adequate aeration. Limited lung volume, increased airway resistance, and decreased compliance result in and add to increased work of breathing, predisposing to respiratory failure. These factors act to limit FRC, and the physiologic implications have been recognized in premature neonates for over half a century. Early reports included vivid descriptions of the increased work of breathing observed in the premature infant with RDS (13). Multiple solutions were proposed to stabilize the chest wall. Many of these are no longer in use today, including negative pressure ventilators (14) and continuous negative pressure boxes (15), or sternal traction (13). In 1971, Gregory reported that continuous positive airway pressure (CPAP) via endotracheal tube or head box increased survival in spontaneously breathing neonates with RDS (16). Innovative ways to deliver CPAP were reported, including face mask, face chamber, pneumask, and nasal prongs (17, 18). The use of CPAP became so extensively studied, that it was claimed that “no new technique in the treatment of hyaline membrane disease has so thoroughly been researched and evaluated as CPAP” (19). However, many limitations of non-invasive support were noted, including air leak (e.g., pneumothorax), need for escalation of support (CPAP failure), and an inability to treat apnea. The combination of these limitations and the advent of ventilators specifically designed for neonates, led to increased use of IMV to treat RDS (19, 20). However, even in this climate, clinical data supported the use of NIV to prevent lung injury in high risk neonates. In 1987, it was reported that very low birth weight infants treated at Columbia University had significantly lower rates of BPD when compared to seven other similar centers in the US (21). Many potential reasons of this finding were considered, including the early and aggressive use of CPAP at this institution. Although no data from randomized trials existed, other clinical reports supported the hypothesis that routine use of NIV decreased the risk of developing BPD (4, 22). Despite these data, studies directly comparing CPAP to IMV as primary support for preterm neonates were not performed until relatively recently.

## Advent of Exogenous Surfactant and its Impact on the Practice of Respiratory Support

It can be argued that the advent of exogenous surfactant in RDS treatment delayed significant refinements in the use of NIV for the early respiratory support of the premature infant. Beginning in the late 1980s, investigators began reporting the results from randomized trials that convincingly demonstrated that the use of “early rescue surfactant” decreased air leak and improved survival in preterm infants with RDS (23). Practice evolved, and results from multiple randomized-controlled trials (RCTs) further refined surfactant therapy. Strategies referred to as “prophylactic surfactant use” or “early rescue surfactant” were proved to reduce air leak and mortality in infants at highest risk of developing RDS (24–26). Thereby, “rescue treatment” was generally defined as surfactant given to intubated patients after RDS had been diagnosed, whereas “prophylactic surfactant” was defined as surfactant given during the initial resuscitation. Findings led to the adoption of these practices as the standard of care for the prevention and treatment of RDS in the US and Europe from the 1990s onward (27).

## A “New Generation” of Preterm Infants and Their Special Need for Respiratory Support

In 2020, preterm infants at highest risk of BPD are different from those enrolled in the surfactant trials in the 1980s and early 1990s. Data collected at the NICHD Neonatal Research Network centers on 34,636 infants between 22 and 28 weeks' gestation between 1993 and 2012 showed that survival increased in those born at 23, 24, 25, and 27 weeks' gestation (3). Data from this same registry demonstrated that rates of BPD seemed to increase in the same population, with rates ranging from ~40 to 90% (3). Thus, it appears that the most vulnerable babies are surviving at rates higher than ever before, but with significant morbidities. It is likely that a major contributor to this improved survival is the enhanced use of antenatal corticosteroids (ACS), having been increased from 24% in 1993 to 87% in 2010 (3).

Both increased survival of the most premature, most vulnerable infants and the increased use of ACS make application of the findings of surfactant trials published in the late 1980s and early 1990s difficult. The infants enrolled in these trials were more mature. For example, the babies enrolled in the surfactant replacement therapy for severe RDS by the Collaborative European Multicenter Study Group were on average 28.5 weeks' gestation (28). ACS exposure was not reported (28). Meta-analyses revealed that babies enrolled in RCTs evaluating the use of prophylactic surfactant were ~27 weeks of gestational age (GA), and ACS exposure was low (~30–40%) (25). Of note, one trial comparing prophylactic vs. rescue surfactant did report a protective effect in the subgroup of babies <26 weeks' gestation; however, ACS exposure was ~30% (29). Undoubtedly, these trials demonstrated that with true surfactant deficiency, preterm neonates need, and respond to exogenous surfactant.

Meanwhile, increased survival of infants at highest risk of BPD and the standardized exposure of these neonates to ACS have driven new clinical questions. Specifically, could it be hypothesized that the respiratory instability demonstrated by this patient population has less to do with primary surfactant deficiency, but more to do with chest wall instability and the inability to recruit, and maintain FRC (30)? And if that were true, should the approach to managing these high risk patients further evolve? Three RCTs comparing routine use of early nasal CPAP with routine intubation and surfactant have been performed: COIN (31), SUPPORT (32), and the *Vermont Oxford Network Delivery Room Management Trial* (VON-DRM) (33). Direct comparison of early CPAP and prophylactic surfactant was only done in the SUPPORT and VON-DRM trials (32, 33), while babies randomized to intubation did not routinely receive surfactant in the COIN trial (31). Importantly because these trials recruited patients antenatally the use of ACS was high (>90%) in both studies. Routine use of CPAP has been shown to be superior to routine intubation and prophylactic surfactant in preventing the combined outcome of BPD or death (10). Other meta-analyses that include a control group not limited to strictly routine intubation and prophylactic surfactant have been published (34, 35). Data from multiple meta-analyses point to a protective signal with routine use of early CPAP preventing lung injury in high risk infants, with a number needed to treat of 17.7 (10), 25 (35), and 35 (34). Current European and US American guidelines recommend prophylactic CPAP and early selective surfactant over primary intubation, prophylactic surfactant and subsequent IMV in preterm infants with RDS (36, 37).

## Non-Invasive Support Failure and Strategies to Prevent it

Knowing that exposure to IMV is as major contributing factor to neonatal lung injury, it is somewhat disappointing that routine use of non-invasive support does not result in a larger treatment effect. One possible explanation may be given by the high rate of CPAP failure (10, 38). Data from both RCTs and observational reports demonstrate that within the first week of life, ~50% of infants initially supported with CPAP require IMV (31–33). Moreover, data suggest that a huge number of infants fail early, within the first 8 h of life (31, 39, 40). GA appears to be a strong predictor of failure, with the most immature neonates failing at the highest rates (31, 40, 41). Based on these observations, multiple interventions aiming at optimizing primary non-invasive respiratory support have been studied.

### Sustained Lung Inflation

The first respiratory efforts of term infants deliver a sustained pressure (30–35 cm H_2_O) over a long inspiratory time (4–5 s) to the lung, resulting in the clearance of lung fluid and the establishment of FRC (42, 43). These initial efforts are blunted in the extremely premature infant whose initial course may be complicated by respiratory depression, decreased respiratory muscle strength, and/or surfactant deficiency. Thus, it has been proposed that providing positive pressure (~20–25 cm H_2_O) for a sustained amount of time (5–20 s) may help to clear lung fluid, establish FRC, and prevent NIV failure (43). This approach has been named “sustained lung inflation” (SLI). Several small RCTs in preterm infants have been published examining different SLI levels and durations (44), demonstrating a decreased need for IMV at 72 h (45–47). However, a meta-analysis of four studies found no difference in the rates of BPD, death, or the composite outcome among those infants treated with SLI compared to standard (44). Moreover, in these studies, SLI did not decrease rates of surfactant replacement therapy for RDS (45–47). Recently, the results from the largest RCT performed to date examining the safety and efficacy of SLI in very immature babies born at 23–26 weeks' gestation, the *Sustained Aeration of Infant Lungs* (SAIL) trial, were published (48). This trial was stopped early, after recruitment of 426 of the calculated 600 infants, due to higher rates of death within the first 48 h of life in the SLI group (48). Of note, SLI compared with standard IMV did not reduce the risk of the primary outcome death or BPD (48). The SAIL trial concluded that SLI maneuver should not be performed in extremely premature infants (48).

### Minimally Invasive Surfactant Therapy

Surfactant deficiency has been assumed one major cause of CPAP failure. Alternative techniques of surfactant administration without using an endotracheal tube have been developed, including nasopharyngeal instillation, laryngeal mask placement and aerolization (49, 50). While none of these methods is ready for clinical application, two promising strategies have evolved, combining the positive effects of surfactant and early CPAP: the INtubation-SURfactant-Extubation (INSURE) procedure and less invasive surfactant administration (LISA) or minimally invasive surfactant therapy (MIST), respectively (38, 51–54). Using sedation and a short period of IMV, INSURE comprises intubation, intratracheal surfactant administration, and immediate extubation to CPAP (38, 49, 52). During LISA, a fine catheter or feeding tube is inserted into the trachea of a preterm infant spontaneously breathing on CPAP, and surfactant is administered slowly over several minutes (51, 53, 54). The very similar MIST approach positions a more rigid vascular catheter via direct laryngoscopy but without using a Magill's forceps (55). A meta-analysis of RCTs comparing INSURE with standard intubation followed by surfactant and IMV, reported a reduced need of IMV and reduced risk of BPD in INSURE cohorts (52). Studies comparing prophylactic INSURE with early CPAP found no benefit of INSURE over CPAP (33, 39). Two meta-analyses documented that prophylactic INSURE did not result in higher survival without BPD (26, 56). Of note, in a retrospective cohort study in 322 preterm infants <32 weeks' gestation who had undergone INSURE, 60% of study infants could not be extubated within 2 h after the procedure (57).

LISA procedure was first described in the early 1990s and was rediscovered about 10 years later (51, 58). It has been widely used in Germany and increasing parts of Europe meanwhile, and is the most intensively studied method of less invasive surfactant therapy (49, 53, 54, 59). The first RCT of the German Neonatal Network including 220 preterm infants born at 26–28 weeks' gestation demonstrated a reduced need of IMV at any time and reduced median days on IMV in the LISA cohort (60). A multi-center study from the same Network in 1,103 neonates <32 weeks' gestation found lower rates of IMV and BPD following LISA (61). So far, seven RCTs have evaluated the efficacy and safety of LISA, with four trials comparing LISA with INSURE (62–64), and three trials comparing LISA with intubation and standard surfactant (60, 65, 66). Two meta-analyses covering these RCTs found a reduction in CPAP failure, need of IMV at any time and a reduction in death or BPD in LISA cohorts (67, 68). It is worth mentioning that the studies included in these meta-analyses were quite heterogeneous. Some of the included trials compared LISA to INSURE (meaning study groups differed solely in the technique of surfactant administration), while some trials compared LISA to standard intubation and subsequent IMV (meaning study groups differed in the approach of both surfactant administration and respiratory support). A more recent meta-analysis attempted to control for study heterogeneity by performing two analyses: one strictly comparing LISA and INSURE, and another comparing LISA to standard intubation and subsequent IMV. LISA was not found to be superior for decreasing BPD or the combined outcome of BPD or death (59). Of note, all RCTs evaluated are small, with only ~450 preterm infants included across all studies. They further differ in risk of bias assessment and study cohorts, ranging from very immature preterm infants in two studies (60, 65) to moderate (63, 64) and late preterm infants in other trials (62, 66). Of note, a comprehensive meta-analysis comprising 30 trials and ~5,600 preterm infants <33 weeks' GA evaluated the effect of different NIV strategies, including CPAP, INSURE, LISA, and nasal intermittent positive-pressure ventilation (NIPPV) vs. IMV on the avoidance of death or BPD (50). The use of LISA was associated with the lowest risk of the latter (50). Recently, the largest cohort study comparing LISA with standard surfactant, so far, has been published by the German Neonatal Network, reporting data on 7,533 preterm infants ≤ 28 weeks' gestation and of whom 1,214 infants had been managed with LISA (69). LISA was associated with reduced risk of mortality and BPD and reduced risk of secondary outcome measures, except for focal intestinal perforation (69).

### Nasal Intermittent Positive Pressure Ventilation

NIPPV has been proposed as an alternative approach of non-invasive support, adding time-cycled positive-pressure inflations to a background support of CPAP (70). A recent Cochrane review comparing primary NIPPV to CPAP concluded that NIPPV prevented intubation in preterm infants (71). However, every trial evaluated in this review, except for one (72), stipulated a diagnosis of RDS for inclusion. Thus, whether NIPPV is superior to CPAP to prevent failure of non-invasive respiratory support for the very tiny baby at high risk of lung injury is unknown. A very recent sub-analysis in the subset of extremely low birth weight infants without RDS found that NIPPV did not decrease failure of primary non-invasive support in these high risk infants (73). A Cochrane meta-analysis found NIPPV was superior to CPAP in preventing extubation failure (74). BPD rates did not differ between both study groups except for those infants who had synchronized NIPPV delivered by a mechanical ventilator (74). This raises the question as to whether NIPPV delivered by neurally adjusted ventilator assistance would be superior to other modes.

### Nasal High-Flow Therapy

Nasal high-flow therapy (nHF) constitutes an additional strategy of nasal breathing support in preterm infants at high risk of lung injury. Heated, humidified, blended air and oxygen are delivered via thin nasal cannulae (75). Perceived benefits include increased comfort and reduced nasal trauma. There are some studies describing the use of nHF as primary respiratory support of preterm infants (76). However, a Cochrane review on nHF vs. CPAP for respiratory support in preterm infants reported that zero infants <28 weeks had been randomized to nHF as primary support, thus making any conclusions in this group impossible (77). Since that time, other RCTs evaluating the same issue have been completed, but none of these trials enrolled neonates born <28 weeks' GA (78–80). It can be safely concluded that there are no data supporting superiority of nHF over CPAP for primary support of very premature babies. In fact, there are data indicating that nHF is inferior to CPAP for this indication. Roberts and colleagues enrolled 564 neonates > 28 weeks' GA with RDS to determine if nHF was non-inferior to CPAP in preventing treatment failure evaluated at 72 h (79). The trial was stopped early due to increased treatment failure in the nHF group. Although the subjects enrolled in this trial are not those at highest risk of lung injury, there is little data to suggest that nHF would perform better in a more premature population. Manley and colleagues randomized 303 preterm infants <32 weeks' GA at first extubation attempt to determine if nHF was non-inferior to CPAP in preventing treatment failure evaluated at 7 days post-extubation (81). nHF was reported “non-inferior” even though treatment failure occurred in 34.2% of infants randomized to nHF vs. 25.8% in the CPAP group. Finally, data guiding the use of nHF as a “weaning modality” from CPAP or directing the reduction and escalation of gas flows are lacking.

### Caffeine

The *Caffeine for Apnea of Prematurity* (CAP) trial established that in high risk premature infants, caffeine reduces the risk of BPD and improves long-term developmental outcomes (82, 83). This protective effect could be largely attributed to a significant reduction in the duration of IMV (82, 83). Importantly, the beneficial effects of caffeine were affected by the timing of initiation of therapy. Subgroup analysis of the CAP trial showed that early (<3 days) compared to later (>3 days) initiation of therapy was associated with a greater reduction in the time on ventilation (84). Additional studies have supported the finding that early caffeine reduces the duration of IMV and enhances the protective effect on BPD (85).

Despite its association with reduced exposure to IMV (37), it cannot be definitely concluded that early caffeine improves the success of NIV. A large observational study showed that early (day of birth) compared to late (after the day of birth) initiation of caffeine did not improve rates of CPAP failure (86). Of note, the average GA of infants in this study was 29–30 weeks, and the rate of CPAP failure was ~20%, suggesting that this conclusion may not apply to the more premature neonate at very high risk of failing non-invasive support. Smaller pilot trials have demonstrated that early administration of caffeine induces demonstrable physiologic effects in this cohort. Administration of caffeine in the delivery room improves respiratory effort, and administration <2 h of age results in hemodynamic benefits (87). The longer-term implications are unknown. Data from adequately powered RCTs are needed to determine whether very early caffeine is safe and improves success rates of non-invasive respiratory support (37).

## Association of NIV with Outcome Measures other than BPD and Long-Term Pulmonary Outcome

Respiratory support of very premature infants cannot be evaluated solely for the prevention of BPD. Other outcome measures, including high-grade intraventricular hemorrhage, necrotizing enterocolitis, patent ductus arteriosus, severe retinopathy of prematurity, and postnatal corticosteroid treatment, were assessed in the COIN, SUPPORT and VON-DRM trial (31–33). Infants treated with early CPAP compared with infants managed with elective intubation and IMV did not significantly differ in any of these outcomes (31–33).

There is growing evidence of persistent pulmonary morbidity in BPD survivors even in the post-surfactant era (2, 88, 89). However, BPD diagnosis does not necessarily predict long-term lung function (89–91). Vice versa, a high incidence of respiratory morbidity has been described in children born preterm, even in the absence of BPD (89, 90). Recent longitudinal cohort data found similar or worse lung function at 8 years follow-up in children born preterm in 2005 compared with cohorts born in 1991 and 1997 (88). Given the increasingly established use of NIV in the more recent cohort of infants, this finding raises the question of long-term effects of NIV. So far, this issue has been addressed in only few prospective studies. The *Breathing Outcomes Study*, follow-up study of the SUPPORT trial, found fewer episodes of wheezing, acute respiratory illnesses and physician or emergency room visits for breathing problems in the CPAP group as compared to the intubation/surfactant group at 18–22 months corrected age (90). Improved lung mechanics and decreased work of breathing at 8 weeks corrected age were reported in a subcohort study of the COIN trial (92).

## Conclusion and Outlook

Non-invasive respiratory support of very immature preterm infants constitutes a paradigm shift—aiming at the prevention of BPD. Current data suggest that composite measures including (i) initiation of CPAP within the first minutes of life, (ii) its continuous delivery at safe and appropriate levels as well as, (iii) targeted surfactant therapy in the spontaneously breathing infant identified with surfactant deficiency may be key to improved success of primary NIV in this cohort. However, published trials have several limitations and future RCTs are necessary. In terms of LISA, the total number of infants covered in existing RCTs is small, and potential adverse side effects still need to be critically reviewed. Further studies are needed to determine the cohort of preterm infants that might benefit most from LISA. NIPPV may offer advantages over CPAP in terms of intubation rates. Scarce data, so far, do not sufficiently back superiority of NIPPV over CPAP and do not support its routine use in very premature preterm infants. Minimal data exist to support the use of nHF as primary mode of support in preterm infants <28 weeks. Evidence of beneficial effects of advanced NIV strategies, such as synchronized modes of NIPPV or nasal high frequency oscillatory ventilation mainly derive from small, single-center studies, differing in patient population, ventilator settings and mode of synchronization, and need to be further studied. Attention needs to be paid to the complex interplay of NIV with other morbidities of prematurity. Given the shortcomings of BPD as a surrogate for long-term pulmonary dysfunction, long-term follow-up, and longitudinal assessment of pulmonary morbidity is required to conclusively determine the impact of NIV on pulmonary outcome later in life.

Future approaches, most likely, will represent a bundle of procedures supporting spontaneous breathing in the very immature preterm neonate. In this context, early initiation of caffeine and optimized caffeine therapy may be vital as adjunctive therapy to prevent apnea and non-invasive support failure.

## Author Contributions

KG and CW wrote the first draft of the manuscript. KG, CS, and CW wrote sections of the manuscript. All authors contributed to manuscript revision, read, and approved the submitted version.

### Conflict of Interest

CS has a consultancy agreement with Chiesi Farmaceutici S.p.A. (Parma, Italy). The remaining authors declare that the research was conducted in the absence of any commercial or financial relationships that could be construed as a potential conflict of interest.
